# Metataxonomic investigation of the microbial community in the trachea and oropharynx of healthy controls and diabetic patients using endotracheal tubes

**DOI:** 10.1371/journal.pone.0259596

**Published:** 2021-11-05

**Authors:** Sun Young Cho, Jeong-Hyun Choi, Seung Hyeun Lee, Yong-Sung Choi, Sung Wook Hwang, Young Jin Kim

**Affiliations:** 1 Department of Laboratory Medicine, Kyung Hee University Hospital, Kyung Hee University School of Medicine, Seoul, Republic of Korea; 2 Department of Anesthesiology, Kyung Hee University Hospital, Kyung Hee University School of Medicine, Seoul, Republic of Korea; 3 Division of Pulmonary and Critical Care Medicine, Department of Internal Medicine, Kyung Hee University Hospital, Kyung Hee University School of Medicine, Seoul, Republic of Korea; 4 Department of Pediatrics, Kyung Hee University Hospital, Kyung Hee University School of Medicine, Seoul, Republic of Korea; 5 Department of Anesthesiology and Pain Medicine, Kyung Hee University Hospital, Seoul, Republic of Korea; University of Illinois Urbana-Champaign, UNITED STATES

## Abstract

**Background:**

Although the study of respiratory microbiota has been an active field of research, obtaining the appropriate respiratory samples for healthy controls remains to be a challenge. As such, this study aims to evaluate the use of endotracheal tube washing as a viable control for sputum samples.

**Methods:**

A total of 14 subjects, including 8 healthy respiratory controls and 6 diabetic patients without any respiratory disease, were enrolled in this study, during which the endotracheal tubes used in their scheduled routine surgery were collected. Pre-operative oral gargles were also collected from non-diabetic subjects.

**Results:**

16S amplicon sequencing revealed similar taxa composition in endotracheal tube washings and oral gargles in the healthy control subjects, although the relative abundance of 11 genus level operational taxonomic units was significantly different between the two sample sources. The diabetic subjects showed relatively lower diversity than those of non-diabetic subjects. The proportion range of the most abundant taxa detected in each endotracheal tube washings were 10.1–33.2%.

**Conclusion:**

Endotracheal tube washing fluid may provide healthy control samples for upper respiratory investigations without incurring any additional risk to the subject.

## Introduction

Next-generation sequencing (NGS)-based microbial community analysis has enabled the unbiased investigation of microbiota, including both culturable and unculturable bacteria [1]. Human microbial community studies using NGS have been widely performed on the gut, skin, vagina, oral cavity, and other non-sterile sites, for better understanding of a microbiologically healthy state and various pathologic conditions [2]. These studies have shown that the infection of specific organs and the host’s overall condition were associated with changes in the microbiota and some researchers have suggested that the study of the microbiota may be helpful in understanding the nature of various diseases, such as infection, metabolic disease, and even malignancies [2, 3].

Compared to other organs, the study of the respiratory microbiota had a delayed start because of the past belief that the lung is an aseptic organ. However, the respiratory tract is currently an active field of research [4–6]. Through respiratory microbiome research, our understanding of respiratory diseases such as asthma, chronic obstructive pulmonary disease, pulmonary fibrosis, upper and lower respiratory infection and lung cancer is currently expanding [7–9]. Furthermore, studies have found that the indices from microbiome analysis in respiratory tract disease can be utilized as clinically useful markers [10, 11].

However, obtaining healthy respiratory control samples in this field of research remains difficult. While a healthy lung tissue can be an ideal specimen, this is rarely available in many hospitals. Furthermore, bronchoalveolar lavage (BAL) fluid and protected specimens brush (PSB) would require invasive techniques, exceeding minimal risk for healthy volunteers [12]. Also, while an induced sputum could reflect the upper area of the lower respiratory tract, its acquisition would still require physical manipulation. As such, healthy respiratory specimens are very difficult to obtain for research purposes. Nevertheless, pathologic respiratory specimens are frequently obtained for diagnostic purposes in clinical laboratories. Among them, sputum tends to be the most common, non-invasive clinical respiratory specimen. However, as healthy people do not normally produce sputum, a corresponding control is difficult to obtain [12]. For this reason, there are currently qualitative and quantitative limitations to the clinical interpretation of NGS data from a sputum specimen. In the context of the microbiota where a bacterial infection is suspected, it is therefore difficult to utilize sputum as a viable sample. Moreover, data on the risk groups that could potentially develop respiratory infections would also be unavailable in this setting.

The aim of this study is to produce healthy control data pertaining to the bacterial community in the tracheal-oropharynx with an endotracheal tube (ETT), which can be used as a control in sputum studies that involve pathological conditions such as bacterial pneumonia. Subsequently, we suggest that the use of ETT washing could be a suitable sampling method for obtaining healthy control samples without causing additional harm to the subject. Furthermore, we also investigate samples from diabetic patients that which represent an infection-free risk group to show that ETT washing can be used for studies on the relationship between non-infectious diseases and the respiratory microbiome.

## Materials and methods

### Healthy respiratory control group

Subjects in the healthy respiratory control group (HC) composed of adult patients who were scheduled for relatively short, up to 3 hours long elective surgeries that required general anesthesia [13]. For pre-anesthesia evaluation, the patients underwent chest radiography, pulmonary function test, blood tests including white blood cell (WBC) count, hemoglobin, AST, ALT, albumin, glucose, and HbA1c, and anti-human immunodeficiency virus (HIV) antibody tests. If there was severe anemia (hemoglobin below 80g/L) [14] or any other abnormal findings from laboratory tests in hematology or respiratory symptoms, the patient was excluded from the control group. The exclusion criteria also included: history of respiratory disease, including tuberculosis or pneumonia; any abnormalities in chest radiography or the pulmonary function test; dental or oral inflammation; autoimmune disease; immunosuppressive treatment; antibiotic use within 3 months; smoking within 3 years; fever; and post-operative pulmonary complications. Finally, while there were no restrictions on height and weight, the patients had to fast for more than 8 hours prior to the surgery.

At 10 minutes before the start of surgery, a prophylactic antibiotic agent was administrated for applicable patients. The ETTs (Covidien, Dublin, Ireland) were collected once the surgery was finished. The depth of intubation was 23 cm for men and 21 cm for women. Using sterile scissors, 15 cm from the end of the tube was cut and placed in a 50 mL conical tube (Neurex life science, Seoul, Korea), washed with 30 mL Diethyl pyrocarbonate (DEPC) treated sterile filtered water with vortexing. Pre-anesthetic gargle, following a 15 s gargle with 15 mL saline, was also collected in order to verify any differences between the ETT wash fluid and the oral gargle. Subsequently, 10 mL of washed fluid was centrifuged at 2,090 x *g* for 15 min at 4°C and the supernatant was discarded. Prior to DNA extraction, sediments and oral gargle were stored at –80°C, no later than 60 minutes since they were collected.

### Diabetic group (DM)

The diabetic group (DM) included patients with type 2 diabetes defined by 6.5% or higher hemoglobin A1c. They were also scheduled for elective surgeries under general anesthesia. With the exception of no restrictions on body mass index (BMI), AST, or ALT, the selection criteria were the same as those for the HC group. For this group, only the ETT specimens and no oral gargle were collected.

### Negative controls

In the operation room, unused ETTs were rubbed on the laryngoscope in a way similar to the intubation procedure and placed in a 50 mL conical tube to be washed with DEPC-treated sterile filtered water with vortexing. For negative control sample preparation, 15 mL of this DEPC water was used. To meet the minimum DNA QC criteria (0.1 ng/μL) for amplicon library preparation, *Vibrio fluvialis* was used for negative control mock sample preparation. This organism was selected in light of the following criteria: 1) its genus is not observed in our pilot study using NGS-metataxonomics of ETT washings (unpublished observations); 2) shares >99% sequence similarity to the reference sequence by 16S rRNA V3~V4; 3) it is not a known component of human respiratory tract flora; and 4) it has never been reported as a cause of reagent contamination in previous reports [15]. *V*. *fluvialis* was grown on blood agar and 15 colonies were inoculated into the 15 mL DEPC water using a sterile 1 μL inoculating loop. A total of two independent negative control samples were prepared.

### DNA extraction, amplification, and deep sequencing

Samples were sent to Macrogen Inc. (Seoul, Korea) and analyzed in accordance with the following procedures. First, DNA was extracted by using PowerSoil DNA Isolation Kit (Mo Bio Laboratories, Inc., cat. no. 12888–100, Carlsbad, CA, USA) according to the manufacturer’s instructions. Subsequently, DNA concentration was measured with Quant-It™ PicoGreen™ dsDNA Assay Kit (Invitrogen, Carlsbad, CA, USA). The library was prepared using a two-step amplification process. Primers used for amplification of a ~550 bp fragment of the V3-V4 region of the 16S rRNA gene, including the overhang adapter, were as follows: 341F (5’-TCG TCG GCA GCG TCA GAT GTG TAT AAG AGA CAG CCT ACG GGN GGC WGC AG-3’) and 805R (5’-GTC TCG TGG GCT CGG AGA TGT GTA TAA GAG ACA GGA CTA CHV GGG TAT CTA ATC C-3’). Each reaction contained 12.5 ng of template DNA (2.5 μL, 5 ng/μL), 200 nM of each primer (5 μL, 1 μM), and 12.5 μL 2× KAPA HiFi HotStart ReadyMix (Anachem, Dublin, Ireland). Amplification was performed by using a Bio-RAD T100 Thermal Cycler (Bio-Rad, Hercules, CA, USA) under the following conditions: denaturation at 95°C for 3 min; 25 cycles of 95°C for 30 s, 55°C for 30 s, and 72°C for 30 s; and final extension at 72°C for 5 min.

Amplified products were purified using AMPure XP beads (Beckman Coulter Life Sciences, Indianapolis, IN, USA). After purification, 5 μL of DNA was used for the second amplification step with Nextera XT Index PCR primers (Illumina, San Diego, CA, USA) (F: 5’-AAT GAT ACG GCG ACC GAG ATC TAC AC -[i5]- TCG GCA GCG TC-3’, R: 5’-CAA GCA GAA GAC GGC ATA CGA GAT -[i7]- GTC TCG TGG GCT CGG-3’; where i5 and i7 represent adapter sequences) according to manufacturer’s instructions. Thermocycler conditions were as follows: denaturation at 95°C for 3 min; 8 cycles of 95°C for 30 s, 55°C for 30 s, and 72°C for 30 s; and final extension at 72°C for 5 min. Purification was performed as before. The concentration and quality of the prepared library was assessed by using an Agilent 2100 Bioanalyzer and DNA 1000 chip (Agilent Technologies, Santa Clara, CA). The prepared library was sequenced by using Miseq (Illumina, San Diego, CA, USA) under a 600-cycle kit (2 x 300 bp) condition.

All ETTs and oral gargles from HC were analyzed in the same batch while the DM samples were analyzed under the same conditions in separate batches.

### Analysis of sequence data and statistical analysis

After sequencing, a FASTQ file was generated using the Miseq reporter. PhiX control reads were removed using Burrows–Wheeler Aligner (BWA) (Version: 0.7.5a-r405) [16]. The paired-end data for each sample were extracted using FLASH (version 1.2.11) [17] and high-quality sequences of 362–466 bp in length and 120–160 bp overlapping lengths were selected. Low-quality, ambiguous, and chimeric sequences were removed using CD-HIT-OTU [18], and the remaining sequences were clustered into operational taxonomic units (OTUs) at a similarity of 97% or more. Taxonomic assignments for each OTU were conducted by using the BLASTN algorithm [19] based on the reference database (National Center for Biotechnology Information 16S rRNA Microbial Database, accessed 31 Dec 2017) and the taxon with the highest identity ratio of 95% or above was assigned. Alpha rarefaction curves and Chao1 index were used to verify that sufficient reads were obtained for each sample by using QIIME (Quantitative Insights into Microbial Ecology, version 1.8) [20]. For the paired comparison of relative abundance of taxa between the ET washings and the oral gargle fluid from HC subjects, QIIME-generated OTU tables were further analyzed by using the Linear Discriminant Analysis (LDA) effect size (LEfSe) method on the Huttenhower lab website (http://huttenhower.sph.harvard.edu/galaxy) [21], where the alpha value for both Kruskal-Wallis tests and pairwise Wilcoxon tests was <0.05 and the threshold on the logarithmic LDA score was >2.0.

For the comparison of diversity between the two sample groups (HC and DM), Chao1, Shannon, and inverse Simpson indices were compared by using two-sample T-tests if the data showed a normal distribution in the Shapiro-Wilk Normality Test. Diversity and abundance were analyzed using R statistical software, version 3.1.2 [22]. The diversity indices of HC and DM subjects were calculated together. Principal component analysis (PCA) was performed to compare the microbiota between the ETT washing fluid and the oral gargle in HC and between the ETT washing fluid from HC and DM, respectively. MedCalc version 11.5.1.0 (MedCalc Software, Ostend, Belgium) was used for the comparison of the two groups and the t-test or the Mann-Whitney test were used depending on the normality of the value distribution.

### Ethical approval and consent to participate

All experiments were performed in accordance with institutional guidelines and were approved by the Kyung Hee University hospital Institutional Review Board (IRB-2015-10-403). Written informed consent was signed and provided by all participants in the study.

## Results

### Patients and clinical specimens

A total of 14 subjects were enrolled in this study and the relevant specimens were collected from August 2016 to April 2017. The 14 subjects were patients who underwent general anesthesia, including 8 HC and 6 DM. Among the 8 HC subjects, one sample (ETT washing) was found to have insufficient reads in the alpha rarefaction curve analysis and was therefore excluded; the remaining 7 pairs of HC samples were used for further analysis.

The median operation time for the 14 patients was 105 min (range: 10–180 min). There was no significant difference in the surgery time and age between HC and DM (Mann-Whitney test, *P* = 0.1747 and 0.1985, respectively). Fasting glucose and hemoglobin A1c (HbA1c) of the 6 DM subjects were 206.5 mg/dL (139–222) and 6.95% (6.7–7.5), respectively.

The clinical characteristics of the patients included in the study are summarized in Table 1 while a detailed clinical information, including the prophylactic antibiotic agent used, is recorded in S1 Table.

**Table 1:** Subject clinical information.

Group	Subject ID	Sex	Age	Diagnosis/Operation	Operation time (min)	BMI	Smoking	HbA1c (%)
**Healthy respiratory control group**	HC01	F	54	Breast cancer T2N0M0/Subtotal mastectomy	110	24.8	Never	6.2
	HC02	M	58	Spinal fracture/Spine pin removal	105	26.6	Stopped for 15 y	5.3
	HC03	F	57	Traumatic avascular necrosis of femoral head/Total hip replacement	120	24.3	Never	na
	HC04	F	22	Ovarian serous borderline tumor/Laparoscopic salpingo-oophorectomy	85	17.3	Never	5.5
	HC05	F	56	Biceps tendon tear/Arthroscopic repair	105	23.7	Never	na
	HC06	M	25	Biceps tendon tear/Arthroscopic repair	10	21.8	Never	na
	HC07	M	40	Shoulder dislocation/Arthroscopic repair	50	21.4	Never	na
**Diabetic group**	DM01	F	59	Osteoarthritis of the knee, both/Total knee Replacement Rt	125	25.8	Never	6.7
	DM02	M	62	Osteoarthritis of the knee, both/Total knee Replacement Lt	165	29.0	Never	7.0
	DM03	F	56	Chronic calculous cholecystitis/Laparascopic cholecytectomy	40	21.7	Never	7.2
	DM04	M	50	Perforated appendicitis/Laparoscopic appendectomy	120	23.7	Never	6.9
	DM05	F	57	Osteoarthritis of the knee, both/Total knee Replacement Rt	155	27.6	Never	6.7
	DM06	M	49	Herniated intervertebral disc/Hemilaminectomy	65	30.1	Never	7.5

Abbreviations: BMI, body mass index; HbA1c, hemoglobin A1c; T2N0M0,; Rt, right; Lt, left.

### Negative controls

The DNA concentration calculated for a blank sample that was not spiked with *V*. *fluvialis* was <0.05 ng/μL, which was considered negligible. In the two negative control samples spiked with *V*. *fluvialis*, DNA concentrations were 0.14 and 4.03 ng/μL. Only one taxon was identified for both negative control samples, and *Vibrio* and *V*. *fluvialis* were assigned at genus and species level, respectively. The number of reads available per sample was 20,159 and 24,525, and no other OTUs were observed.

### Healthy respiratory control group

The median (range) number of reads from the ETT washings and the oral gargle were 84,835 (43,229–150,822) and 129,549 (100,992–136,853), respectively. The number of OTUs in each sample ranged from 136 to 242 among the 7 healthy controls. A total of 131 genera were assigned.

**Fig 1:**
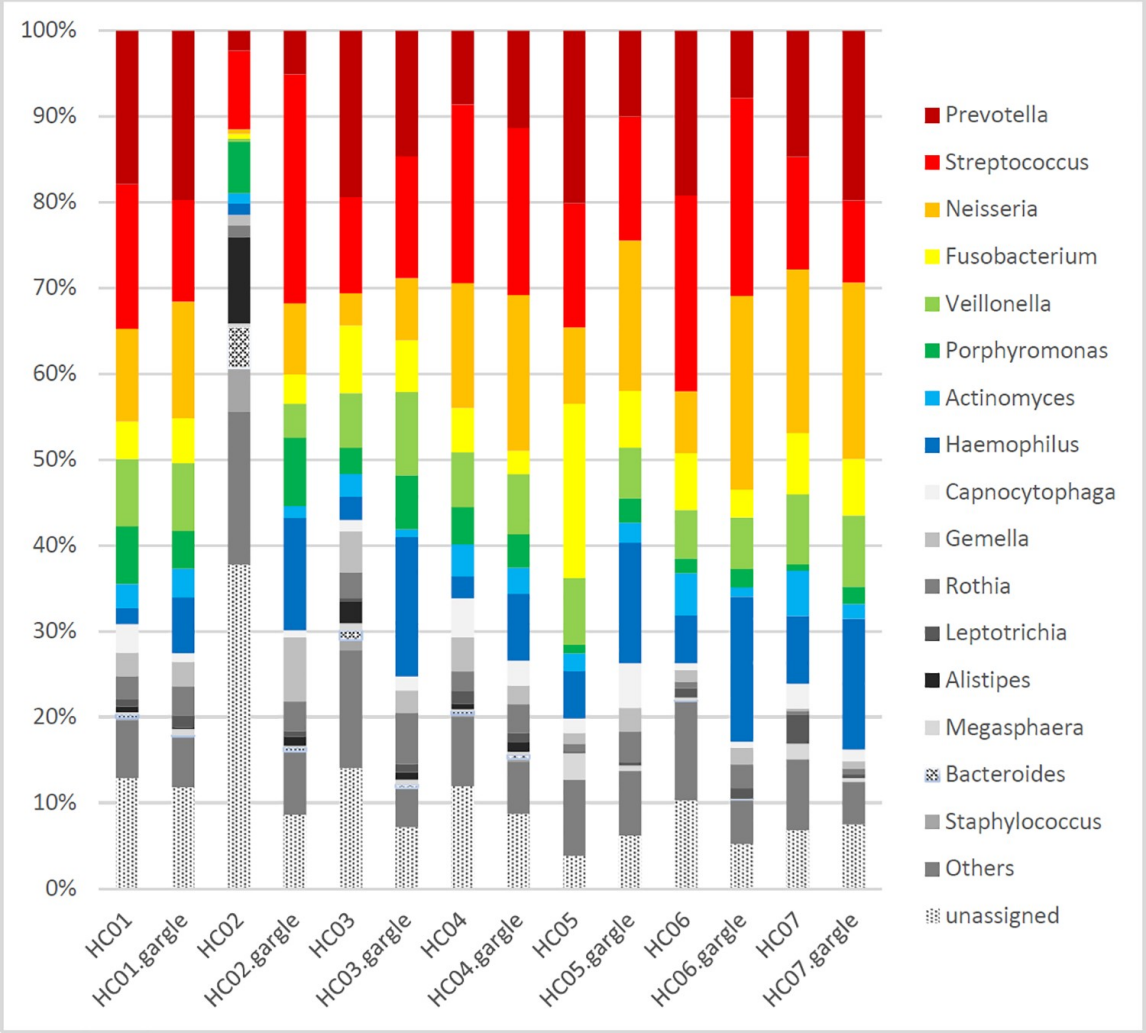
Relative abundance of microbial community constituents in endotracheal tube washings and oral gargles from seven healthy respiratory control group. Taxa that did not show more than 3% abundance in at least one specimen were designated as “others”.

Bacterial OTU distribution at the genus level in the ETT and oral gargle sample pairs from HC subjects are shown in S1 Data and Fig 1, where genera with an abundance of more than 3% are highlighted. The top 10 frequently identified genera and their relative percentage (median, range) in the ETT sample were *Prevotella* (17.9, 2.4–20.1), *Streptococcus* (14.5, 9.1–22.9), *Neisseria* (8.9, 0.6–19.1), *Fusobacterium* (6.6, 0.5–20.3), *Veillonella* (6.4, 0.3–8.2), *Porphyromonas* (3.1, 0.7–6.7), *Actinomyces* (2.8, 1.2–5.3), *Haemophilus* (2.7, 1.3–7.9), *Capnocytophaga* (1.7, 0–4.5), and *Gemella* (1.4, 0.3–4.8). The highest abundance of any genus measured in an individual ETT washing sample was 22.9%, which was observed for *Streptococcus* in sample HC01. Similarly, the most frequently detected genera in oral gargles were *Neisseria* (17.5, 7.2–22.5), *Streptococcus* (14.4, 96–26.7), *Haemophilus* (14.1, 6.5–16.9), *Prevotella* (11.3, 5.1–19.8), *Veillonella* (7.0, 3.9–9.7), *Fusobacterium* (5.3, 2.7–6.6), *Porphyromonas* (3.9, 2.0–8.0), *Rothia* (3.4, 0.7–6.0), *Gemella* (2.6, 0.9–7.5), and *Actinomyces* (1.7, 0.9–3.3). The highest proportion of both *Alistipes* assignment and unassigned OTUs occurred in HC02, at 10.1% and 37.8%, respectively.

### Difference between endotracheal tube washing and oral gargle

**Fig 2:**
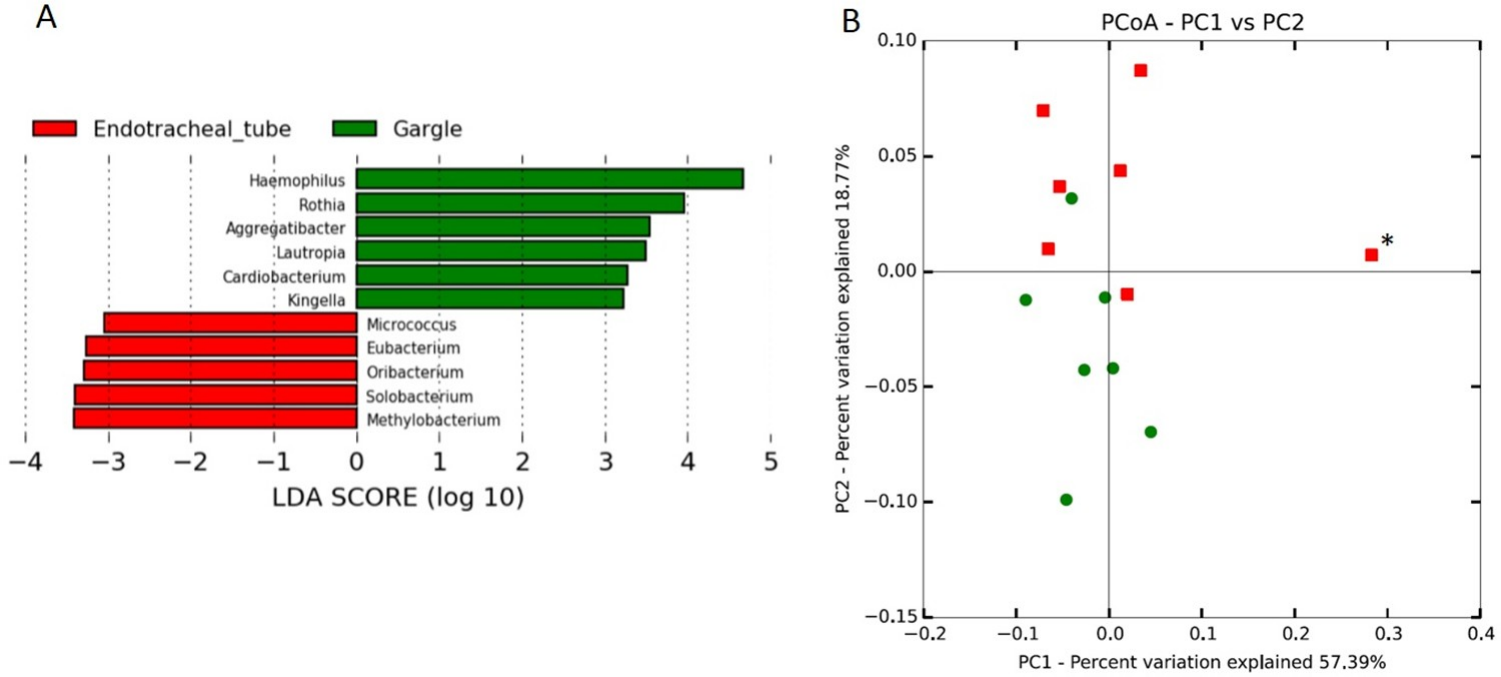
Comparison between ET washings and oral gargles. (A) Comparison of relative abundance of genera between ET washings (red) and oral gargles (green). The 11 genus level OTUs gave linear discriminant analysis (LDA) values > 2. (B) PCA plot for ET washings (red) and oral gargles (green). *indicates sample HC02.

Metataxonomic analysis revealed statistically significant differences in taxa abundance between the ETT washing and oral gargle samples. In the gargle samples, 6 OTUs (*Haemophilus*, *Rothia*, *Aggregatibacter*, *Cardiobacterium*, and *Kingella*) were more abundant whereas 5 different OTUs (*Micrococcus*, *Eubacterium*, *Oribcterium*, *Solobacterium*, and *Methylobacterium*) were more abundant in the ETT samples (Fig 2A). PCA was conducted in order to compare the correlation of the microbial community structure across each sample. The result suggested that the microbial community structure of most samples was similar except for HC02. This was supported by the values for variance explained by PC1 and PC2, which were 57.39% and 18.77%, respectively (Fig 2B).

### Diabetic patients

**Fig 3:**
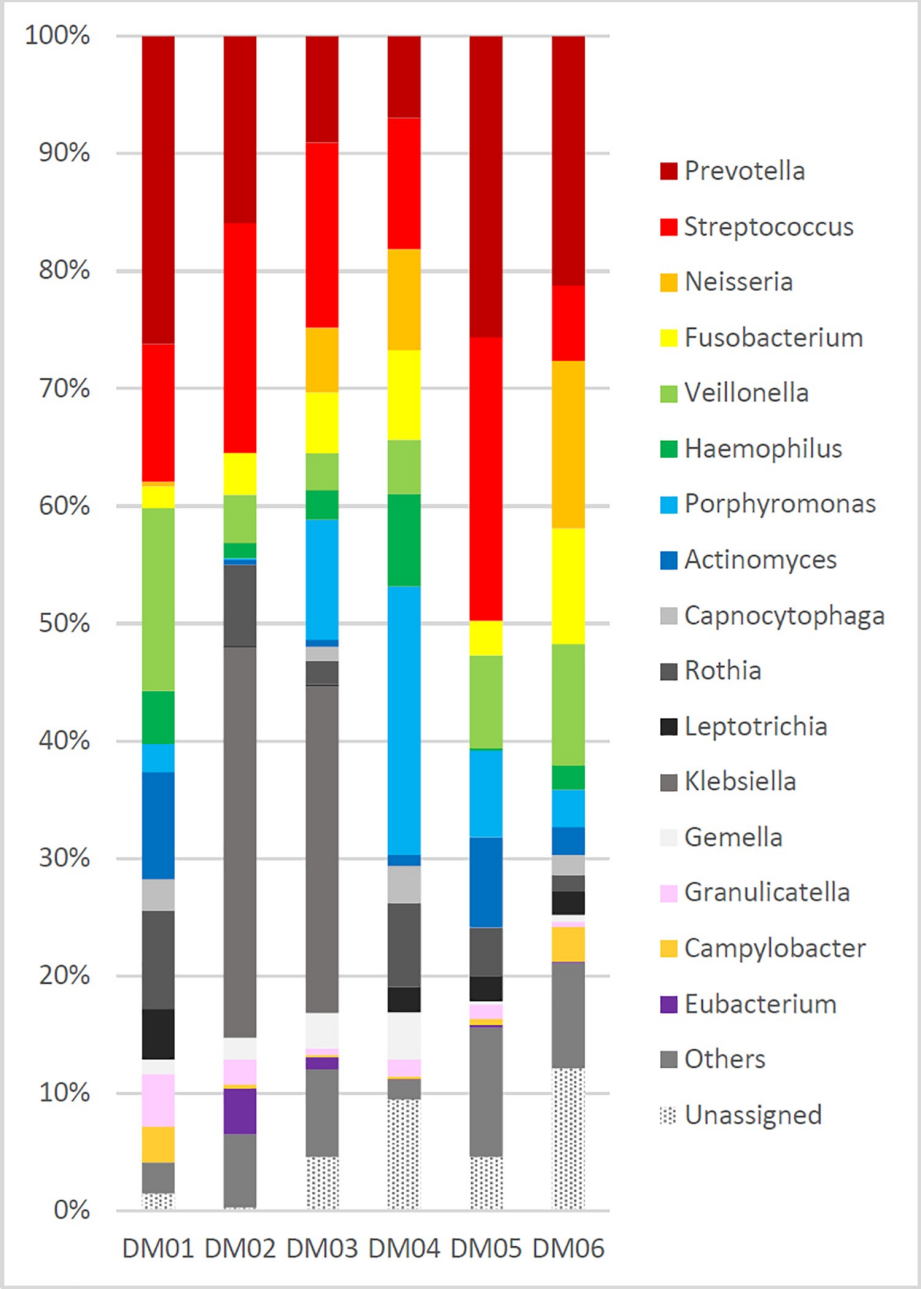
Relative abundance of microbial community constituents in endotracheal tube washings from six diabetic patients. Taxa that did not show more than 3% abundance in at least one specimen were designated as “others”.

The median (range) number of reads from the ETT washings of DM subjects was 40,128 (34,051–125,389) and the number of OTUs in each sample ranged from 90 to 173 (Fig 3 and S2 Data). A total of 125 genera were assigned, where the top 10 and their relative percentage (median, range) were *Prevotella* (18.6, 7.0–26.2), *Streptococcus* (13.7, 6.4–24.1), *Veillonella* (6.3, 3.1–15.6), *Rothia* (5.5, 1.4–8.4), *Porphyromonas* (5.3, 0.1–22.9), *Fusobacterium* (4.4, 1.9–9.8), *Neisseria* (2.9, 0–41.2), *Haemophilus* (2.3, 0.2–7.9), *Leptotrichia* (2.1,0.1–4.3), and *Actinomyces* (1.3, 0.4–9.1). *Klebsiella* was the dominant genus in both DM02 and DM03, at 33.2% and 27.8% relative abundance, respectively.

**Fig 4:**
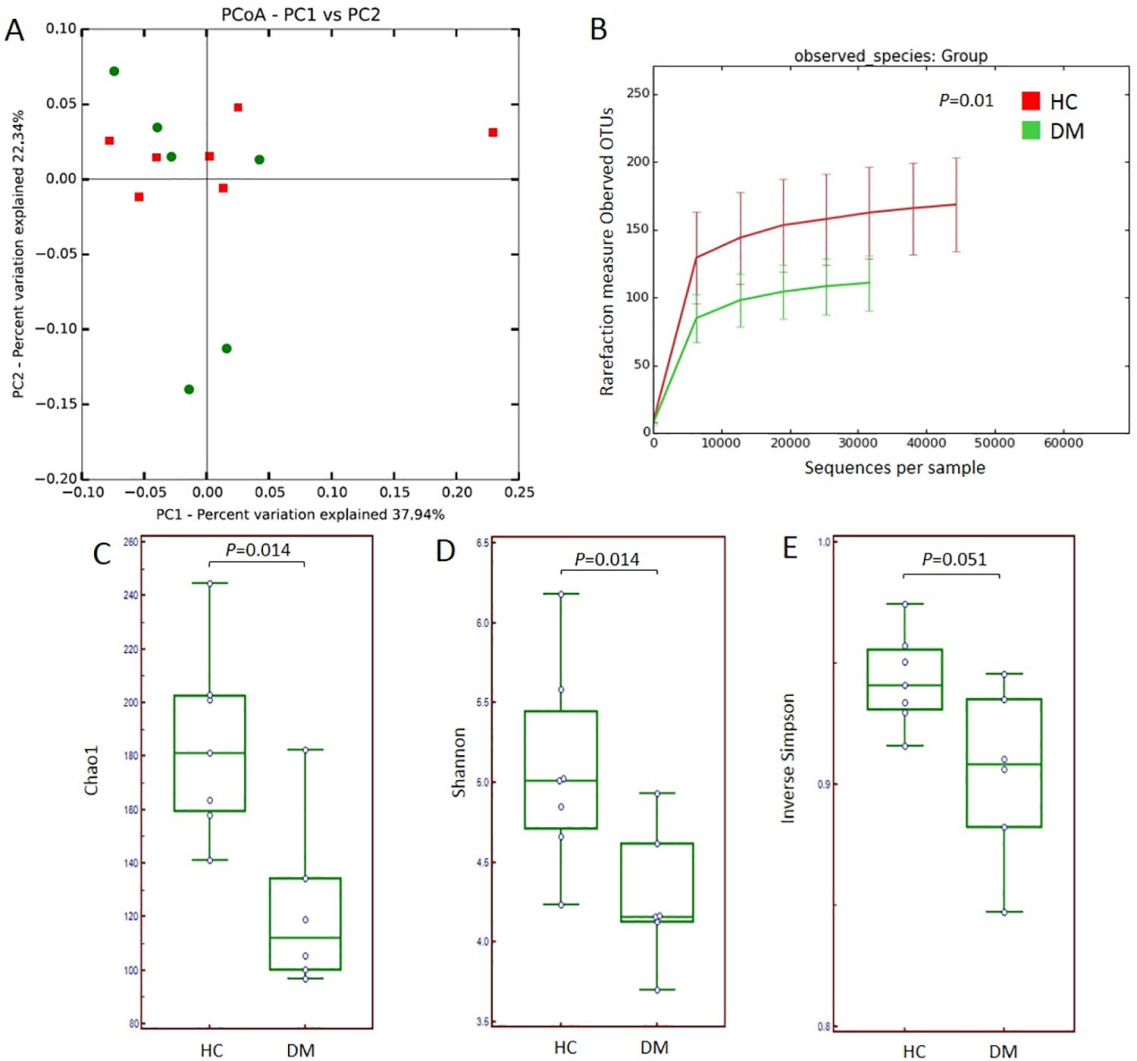
Comparison of variation and diversity indices between healthy respiratory control (HC) and diabetic subjects (DM). (A) PCA plot for HC (red) and DM (green) (B) Rarefaction curve based on observed OTU by sampling group. (C) Chao1 box plots. (D) Shannon box plot. (E) Inverse Simpson box plot.

The percentage variance explained for each axis (PC1 and PC2) in PCA were 37.94% and 22.34%, respectively (Fig 4A). The median number of OTUs for DM and HC were 109 and 169, respectively, where the number in DM samples was significantly lower than in HC samples (Mann-Whitney test, *P* = 0.010) (Fig 4B). Diversity indices, Chao1 (*P* = 0.014) and Shannon (*P* = 0.014), were significantly lower in DM samples than HC samples (Mann-Whitney test) while the Inverse Simpson index showed a *P* value of 0.051 (Table 2 and Fig 4C–4E).

**Table 2:** Diversity comparison between healthy respiratory controls and diabetic subjects.

Diversity Index	DM		HC		*P* value
	Median	Interquartile range	Median	Interquartile range	
OTUs	109.0	97.0–128.0	169.0	147.5–195.8	0.014
Chao1	112.2	100.3–134.4	181.4	159.4–202.6	0.014
Shannon	4.161	4.130–4.619	5.015	4.709–5.445	0.014
Inverse Simpson	0.941	0.0.883–0.935	0.908	0.931–0.956	0.051

Abbreviations: OTU, operational taxonomic unit; DM, diabetic group; HC, healthy respiratory control group.

## Discussion

Studies of the healthy human respiratory tract microbiota frequently use specimens such as BAL, PSB, endotracheal aspirates, and specimens obtained from organs adjacent to the respiratory tract, such as nose and mouth [23–25]. Sputum is the most commonly used respiratory specimen in clinical laboratories, and it is widely used for research in respiratory diseases [26, 27]. However, due to the lack of an optimal corresponding healthy control sample for sputum, induced sputum has been collected from healthy volunteers as a control group [28]. In light of this, we used ETTs to obtain healthy control samples for a respiratory bacterial community study. The ETT is tightly in contact with the tracheal wall by means of a balloon cuff, and the extubation pathway is identical to that of the sputum. These specimens can be easily obtained from the medical institutions where surgery is performed, and there is no risk of additional harm to the patients arising from participation. We demonstrated that the ETT washing and oral gargle samples have different taxonomic abundance ratios, suggesting that the former leads to an acquisition of respiratory samples that are different from the later. However, we also found overall consistency over the microbiota (i.e., microbial community structure) between sample groups (see Fig 2B), supporting previous studies that show the similarity between mouth and lung microbiota [23].

In clinical disease research, the inclusion of healthy controls and risk groups can help in terms of yielding meaningful results. Here, we used type 2 diabetic patients as a risk group for pneumonia. We did not set limitations on weight, aspartate transaminase (AST), or alanine aminotransferase (ALT) for the selection of the diabetic group because these values are frequently elevated in diabetic patients [29]. Although the microbiota between DM and HC samples were similar in PCA, the diversity indices based on OTU calculations were lower in DM samples than in HC samples (Fig 4C–4E). Diabetes is characterized by considerable morbidity and mortality, mainly from cardiovascular and renal complications, and it is well known that diabetes predisposes to a variety of infectious diseases, including pneumonia [30]. Studies have also shown that the lungs can be a target of diabetic complication where related pathologic findings include inflammatory cell infiltration and increased fibrosis [31, 32]. In a study using mice with DM, it was reported that DM-related dysbiosis of gut-lung axis activates the NF-κB signaling pathway that leads to fibrotic changes in the lung tissue [31]. However, there are few studies on DM and airway dysbiosis and therefore further research on the respiratory microbiome and the pathologic changes in the airway would be necessary to better understand the impact of DM on the respiratory microbiome [33]. The impact on the clinical status of each patient, in addition to diabetes, should also be considered in order to interpret the results of the diabetic patients who demonstrated lower diversity values than those of HC subjects.

When the microbial community study results are interpreted, reagent and device contamination needs to be taken into consideration [15]. ETT intubation is not generally performed aseptically and therefore there is a risk of contamination. To exclude the contaminant bacterial reads originating from the ETT, laryngoscope, and the sample transport tube, a negative control sample was prepared using the same procedure, except for contacting the patient. For library preparation, *V*. *fluvialis* was added to the negative control fluid in order to meet the quality control criteria. Subsequently, the laboratory *V*. *fluvialis* quality control strain was re-analyzed by 16S rRNA gene sequencing and BLAST search, which demonstrated a 99.73% sequence similarity with *V*. *fluvialis* (GenBank ID: NR_114218.1), followed by *Allomonas enterica* (99.46%, NR_114900.1) and *Vibrio furnissii* (99.46%, NR_037067.1). *V*. *fluvialis*, *V*. *furnissii*, and *A*. *enterica* share >99% 16S rRNA gene sequence similarity [34]. This indicates that their differentiation via the 16S rRNA gene V3-V4 region could be difficult; however, the only genus detected in our *V*. *fluvialis-*spiked negative control sample was *Vibrio*. While this may not suggest that there was no contamination during the experiment, the DNA concentration from any contaminating bacteria was sufficiently low to the extent that it did not affect our results. Nevertheless, in order to use ETT washings for microbiome analysis effectively, physicians performing intubation and extubation must be very careful to minimize contamination.

In HC subjects, the ETT washing fluid and oral gargle samples showed similar distributions of the top 10 most abundant taxa. However, in the H02 sample, the proportion of ‘unassigned’ and ‘others’ in the ETT washing fluid reached 37.8% and 17.8%, respectively, while the ranges in the other six HC samples were 3.8–14.1% and 6.8–13%, respectively. The abundance of *Alistipes* in HC02 (10.1%) was the highest among all samples, and the OTU distribution in the ETT fluid was different from in the oral gargle obtained from the same patient (1.1%). This was not in line with the existing study investigating the microbiota of mouth and lower respiratory tract, which reported the microbial communities identified on these two sites to be similar [23]. The unique pattern seen in the ETT washing of subject HC02 may be caused by an accidental momentary intubation of the ETT into the esophagus during the procedure. However, it must be noted that similar results have been previously reported in the oropharynx and esophagus [35]. In case of HC02, the intubation was done at once without any mistakes. *Alistipes*, which was observed as a major taxon in HC02, is a component of the human gastrointestinal microbiota [36] However, to the best of our knowledge, it has not been reported as major component of the respiratory tract microbiota in healthy humans. In addition, the OTU to which *Alistipes* was assigned in HC02 demonstrated a 95.9% (396/413) sequence identity with the reference sequence, which is close to the taxon assigning limit used in our study (95%). Furthermore, the proportion of unassigned OTUs in HC02 (37.8%) was higher than in other samples. Taken together, the results from subject HC02 are difficult to interpret clinically and raise suspicions of contamination. In any case, however, this presents a good example of the potential use of oral gargle as a paired quality control sample when analyzing respiratory specimens.

Given the fact that reads with 97% or greater sequence similarity were clustered to the same OTU, we acknowledge that the diversity analysis in our study may underestimate the actual microbial community composition in the genus or species level. *Streptococcus*, which is a major component of the respiratory microbiota, possesses a high 16S rRNA gene sequence homology across different species. In that sense, the diversity may have been underestimated since other species with similar 16S rRNA gene sequences may have been clustered into a single OTU.

In terms of taxon assignment, sequence identity of more than 95% was used to determine genus level designations in our study. The Clinical & Laboratory Standards Institute (CLSI) guideline MM18-A [37], which is used as a reference for the identification of bacteria using 16S rRNA gene sequences in clinical laboratories, suggests that the specified percent identity to reference sequences for genus and species identification are more than 97% and 99%, respectively. The guideline also states that the lowest limit that can be reported as “most closely related to genus” is 95% [37]. We adopted this criterion as a basis for genus-level annotation in our study. However, the guideline was developed mostly for interpreting Sanger sequencing of cultured strains and is not universally applied to NGS-based cluster analysis. Furthermore, the percentage identity criterion for the annotation of OTUs used in previous clinical studies either varied from 80 to 97% or was not described [23, 38–41]. As such, further research would be necessary to suggest the appropriate criteria for clinical microbial community studies.

In a clinical laboratory, it is important to identify organisms at the species level. However, due to high similarity within the genera, it can be difficult to do so for oral and respiratory microbiota such as *Streptococcus*, *Hemophilus*, and *Neisseria* by 300–400 bp amplicon sequencing of the 16S rRNA gene [37]. Studies of pneumonia pathogens also require species level identification. Bacterial community analysis using broader markers such as the full-length 16S rRNA gene and the 16S-23S rRNA gene region may be necessary in clinical studies [42, 43]. In this study, we selected the V3-V4 region in consideration of the longest read length and cost, but there is a limitation in not investigating the difference in results that may occur when other regions are targeted. In Partial 16s rRNA gene sequencing, the difference in results pertaining to the selection of hypervariable regions of the 16S rRNA gene on various clinical samples has been previously reported [44, 45]. For respiratory samples, the V1-V2 region was selected for better species-level taxonomic assignment in a study with endotracheal aspirates and oropharyngeal swab [40]. In another study comparing the V3-V4 and V4 regions, V3-V4 showed better taxonomic resolution than V3, although the difference was not great and protocol bias was related to sample biomass [46]. As the cost of 16s full-length sequencing has been recently decreasing along with technological improvements, related research is expected to increase.

Previous studies of the respiratory microbiota have also used ETTs [40, 47]. Using ETT aspirate samples, they studied the airway microbiota in patients with respiratory failure who need mechanical ventilation. These studies show that decreased diversity and taxa that which corresponds to potential pathogens not identified in culture can be found by 16S amplicon sequencing. What is novel in this study is that we collected ETT tips from patients with normal respiratory function and because of this, it would be difficult to make direct comparisons between our results and those of critically ill patients. Nevertheless, we found that Veillonellaceae, Prevotellaceae, and Streptococcaceae were observed as common major taxa in the healthy control group of our study and previous study [40].

With the exception of HC02, healthy control subjects showed a similar distribution of major taxa in the oral gargle and ETT washing samples where in which the top 10 genera were found across all samples albeit the proportions were somewhat different. Although the human oral microbiota is known to have high interpersonal variation, it has been reported that dominant taxa may show common features [48]. All subject patients fasted for more than 8 h before surgery, and this should be considered when interpreting the similar taxa distribution in the healthy control group as well. Going further, future studies should assess interpersonal variations of the respiratory microbiota in a larger cohort of healthy controls while also analyzing their relationships with resident environment, immunity, nutrition, age, sex, and gender [49]. In such a study, ETT harvesting may serve as a useful sampling method.

In conclusion, ETT washing fluids could be considered as a viable source of control sample in sputum analyses. ETT harvesting allows for the collection of specimens without incurring any additional risk to patients, and therefore the collection of these specimens from patients without respiratory failure that are subsequently paired with clinical information such as DM may be helpful in further studies that focus on the relationship between the human airway microbiota and the relevant pathological conditions.

## Supporting information

S1 Table Detailed clinical information of included patients. (XLSX)

S1 Data Genus level relative abundance of microbial community in the samples including endotracheal tube and oral gargle. Genera with an abundance of more than 3% are highlighted.
(XLSX)

S2 Data Genus level relative abundance of microbial community in the diabetes group. Genera with an abundance of more than 3% are highlighted.
(XLSX)

## Abbreviations

ALT: alanine aminotransferase
AST: aspartate transaminase
BAL: bronchoalveolar lavage
COPD: chronic obstructive pulmonary disease
DM: diabetic patients
ET: endotracheal tube
HbA1c: hemoglobin A1c
HC: healthy respiratory controls
NGS: Next-generation sequencing
OTUs: operational taxonomic units
PSB: protected specimens brush
